# Double-Antigen Lateral Flow Immunoassay for the Detection of Anti-HIV-1 and -2 Antibodies Using Upconverting Nanoparticle Reporters

**DOI:** 10.3390/s21020330

**Published:** 2021-01-06

**Authors:** Iida Martiskainen, Etvi Juntunen, Teppo Salminen, Karoliina Vuorenpää, Sherif Bayoumy, Tytti Vuorinen, Navin Khanna, Kim Pettersson, Gaurav Batra, Sheikh M. Talha

**Affiliations:** 1 Department of Biotechnology, University of Turku, 20520 Turku, Finland; iimama@utu.fi (I.M.); etvi.juntunen@gmail.com (E.J.); tjsalm@utu.fi (T.S.); karoliina.vuorenpaa@gmail.com (K.V.); shebay@utu.fi (S.B.); kimpet@utu.fi (K.P.); 2 Department of Virology and Clinical Microbiology, University of Turku, 20520 Turku, Finland; tytti.vuorinen@utu.fi; 3 International Centre for Genetic Engineering & Biotechnology, New Delhi 110067, India; navinkhanna5@gmail.com; 4 Translational Health Science and Technology Institute, Faridabad 121001, India; gaurav.batra@thsti.res.in

**Keywords:** rapid testing, HIV, lateral flow assay, upconverting nanoparticles, diagnostics, point of care

## Abstract

Rapid diagnostic tests (RDTs) are often used for the detection of anti-human immunodeficiency virus (HIV) antibodies in remote locations in low- and middle-income countries (LMIC) with low or limited access to central laboratories. The typical format of an RDT is a lateral flow assay (LFA) with visual interpretation prone to subjectivity. This risk of misinterpretation can be overcome with luminescent upconverting nanoparticle reporters (UCNPs) measured with a miniaturized easy-to-use reader instrument. An LFA with UCNPs for anti-HIV-1/2 antibodies was developed and the assay performance was evaluated extensively with challenging patient sample panels. Sensitivity (*n* = 145) of the UCNP-LFA was 96.6% (95% CI: 92.1–98.8%) and specificity (*n* = 309) was 98.7% (95% CI: 96.7–99.7%). Another set of samples (*n* = 200) was used for a comparison between the UCNP-LFA and a conventional visual RDT. In this comparison, the sensitivities for HIV-1 were 96.4% (95% CI: 89.8–99.3%) and 97.6% (95% CI: 91.6–99.7%), for the UCNP-LFA and conventional RDT, respectively. The specificity was 100% (95% CI: 96.4–100%) for both assays. The developed UCNP-LFA demonstrates the applicability of UCNPs for the detection of anti-HIV antibodies. The signal measurement is done by a reader instrument, which may facilitate automated result interpretation, archiving and transfer of data from de-centralized locations.

## 1. Introduction

Human immunodeficiency viruses 1 (HIV-1) and 2 (HIV-2) are the etiological agents of HIV infection. These viruses infect cells of the adaptive immune system causing profound immune suppression [1]. This leads to acquired immunodeficiency syndrome (AIDS) if the infection is not diagnosed and medicated at an early stage. Currently, the number of people living with HIV is estimated to be 36.9 million, of which approximately 5% represent new infections on a yearly basis. In 2017, there were 940,000 AIDS-related deaths globally [2]. The vast majority of people living with HIV are in low- and middle-income countries (LMICs), with approximately 66% living in sub-Saharan Africa [2]. It has been estimated that only 79% of people living with HIV are aware of their status. HIV testing is an important part of AIDS prevention since it enables the initiation of the antiretroviral treatment and provides awareness for the patient, which helps to prevent further transmissions [3].

In LMICs and in resource-limited settings, rapid diagnostic tests (RDTs) are commonly used for the diagnosis and screening of HIV infection. RDTs can be used in remote health stations for diagnostic purposes but also in emergency medicine departments and for screening high-risk populations such as men who have sex with men, people who inject drugs, people in prisons and other closed settings and sex workers and their clients [3].

Lateral flow assay (LFA) platform is the dominating RDT format because of the low cost and ease of use. There are several LFAs based on anti-HIV antibody detection available on the market. The typical label technology used in these tests is colored or gold nanoparticles, with a visual interpretation of results which can provide adequate performance for anti-HIV screening. However, the use of visual labels has been associated with the risk of misinterpretation of the results depending on the experience of the interpreter and physical circumstances such as light [4,5,6,7,8,9].

Recently, there has been an interest in replacing the visual labels used in LFAs with luminescent reporter technologies such as upconverting nanoparticles (UCNPs) [10,11,12,13]. The advantages of the UCNP technology are a clear numerical cutoff to determine the status of the patient and improved sensitivity, particularly in case of antigen detection [10,11,12]. Furthermore, UCNP reporter technology has been utilized for the detection of anti-viral antibodies [14]. Compared to traditional fluorescent labels, upconversion luminescence measurement is free from background autofluorescence due to the conversion of lower-energy excitation light into higher-energy emission at visible wavelengths [15] and thus provides potential for use in sensitive applications.

**Figure 1:**
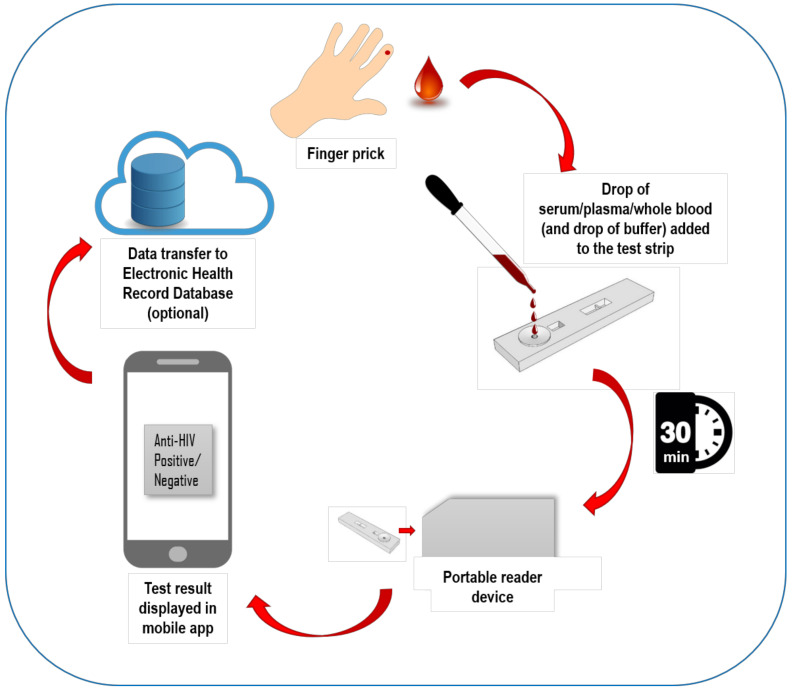
Schematic illustration of the ideal technology components and workflow of the upconverting nanoparticle (UCNP) reporter-based lateral flow assay (UCNP-LFA).

Measurement optics required for UCNP reporters can be provided in a miniaturized and cost-effective format and point-of-care testing (POCT)-compatible UCNP readers have been described in the literature [16,17]. Measurement instrumentation can include ideal next-generation point-of-care testing (POCT) features such as connectivity to cloud, smart applications and portal services and enable access to the POCT results and test history anytime and anywhere [18]. Ideally, a procedure for infectious disease testing such as HIV testing could include use of few drops of finger-prick sample added to the test strip cartridge followed by addition of chase buffer. The signal is read in less than 30 min [19,20] with a portable reader device, the test result can be displayed in a mobile phone-based application and the technology enables the data transfer to electronic health record system (Figure 1).

Based on the interesting properties of UCNP reporters, we developed a UCNP-LFA for the detection of anti-HIV-1 and -2 antibodies. The aim of the study was to show the applicability of the UCNP detection technology for the detection of anti-HIV antibodies and to extensively evaluate the assay performance with challenging patient sample panels.

## 2. Materials and Methods

### 2.1. Clinical Samples and Reference Assays

The following sample panels were purchased from SeraCare Life Sciences Inc. (Milford, MA, USA): Anti-HIV-1 Mixed Titer (0800-0303) (PRB205), Anti-HIV-1 Low Titer (0800-0301), HIV-1 Early Infection Performance (0800-0297), AccuSet HIV-1 p24 Performance Panel (0800-0362), AccuSet HIV-1/2 Performance Panel (0800-0380), HIV-1/2 Worldwide Performance Panel (PRZ206) and a Viral Co-infection Panel (PCA201). Fifty-three disease state samples were purchased from Labquality Oy (Helsinki, Finland), 18 anti-HIV-positive disease state samples were purchased from Biomex GmbH (Heidelberg, Germany) and 100 presumed HIV-negative samples were purchased from Turku University of Applied Sciences (Turku, Finland). Apart from commercially available sample panels, 217 clinical serum and plasma samples tested negative for HIV and 15 samples tested positive for anti-HIV-1 antibodies were obtained from the Department of Virology, University of Turku (Turku, Finland). All the patient data, except for the status for HIV infection, were anonymized and no personal data of the patients were handled. Use of these samples in this study was approved by the Ethical Committee of the Hospital District of Southwest Finland (Decision T012/011/18). The samples used in this study are described in more detail in Table 1. A set of 100 negative and 100 positive samples was evaluated with the commercially available Alere HIV Combo (Abbott Laboratories, Chicago, IL, USA) rapid test.

### 2.2. Bioconjugation of UCNP Reporters

Recombinant HIV-1 and -2 envelope glycoproteins (r-HIV-1env and r-HIV-2env, respectively) were expressed in *Escherichia coli* and purified as described earlier [21]. A 1:1 mixture of r-HIV-1env and r-HIV-2env antigens was covalently coupled to the surface of carboxylated Upcon UCNP reporter particles of 68-nm diameter with a hydrophilic coating (Kaivogen Oy, Finland). UCNP solution was centrifuged for 30 min at 20,000× *g*. The supernatant was removed and the UCNP surface was activated by suspending the pellet into 260 µL of 20 mM MES buffer and 2 mM potassium fluoride (KF), pH 6.1, containing 20 mM EDC (1-Ethyl-3-(3-dimethylaminopropyl)carbodiimide) and 30 mM sulfo-NHS. The activation step was performed at room temperature with rotation for 15 min. The UCNPs were washed by centrifugation for 10 min at 20,000× *g*, removing the supernatant and suspending the UCNPs into 20 mM MES, pH 6.1. The UCNPs were centrifuged as before and the pellet was resuspended into 20 mM MES, pH 6.1, containing 65 µg each of r-HIV-1env and r-HIV-2env antigens per 1 mg of UCNPs. The bioconjugation reaction was incubated for 2.5 h at room temperature with rotation and the reaction was stopped by adding glycine, pH 11, to a final concentration of 50 mM. The reaction was further incubated for 30 min at room temperature with rotation. After this step, the UCNP conjugates were washed twice to remove unbound compounds by centrifugation for 20 min at 17,200× *g* at +4 °C and the UCNP pellet was suspended to 500 µL of storage buffer containing 20 mM Na-carbonate, 0.05% NaN3, 0.05% Tween-85 and 2 mM KF, pH 9.6. This wash step was repeated twice, and finally, the pellet was suspended to 250 µL of storage buffer and bovine serum albumin (BSA) was added to a final concentration of 0.5%.

### 2.3. Preparation of LFA Strips

Nitrocellulose membrane CNPH-N-SS60 (Advanced Microdevices Pvt. Ltd., Haryana, India) with a width of 25 mm was laminated to a backing plastic card (Standard Grade Backing Laminate, Kenosha Tapes, The Netherlands). A cellulose absorbent pad (CFSP223000, Merck Millipore, MA, USA) with a width of 27 mm was used as a wick. A conjugate pad (PT-R1, Advanced Microdevices Pvt. Ltd.) was attached next to the nitrocellulose membrane and a sample pad pre-treated with 10 mM Tris-HCl, 0.2% BSA and 0.1% Tween-20, pH 8.5 (Cytosep 1662, Ahlstrom-Munksjö Oyj, Helsinki, Finland), was attached next to the conjugate pad. The pads were overlapping, by 1–2 mm, the nitrocellulose and each other. The test line containing 1:1 mixture of r-HIV-1env and r-HIV-2env antigens was printed to the nitrocellulose membrane with a density of 0.25 µg/cm total antigens in 10 mM MES pH 6.1 printing solution. Control line HIV-1 gp41 rabbit polyclonal serum (ANT-160, ProSpec-Tany TechnoGene Ltd., Rehovot, Israel) was printed to the nitrocellulose membrane diluted 1:25 in 10 mM Tris-HCl pH 8.0 printing solution. The position of the test line was 12 mm from the edge of the nitrocellulose and the position of the control line was 5 mm from the test line. The line dispensing was done using a liquid dispenser (Scienion AG, Berlin, Germany). After the lines were dispensed, the cards were dried for 30 min at +35 °C. Before use in the assay, the nitrocellulose membrane was protected with transparent cover tape (Kenosha Tapes, Amstelveen, The Netherlands) and 4.8-mm wide strips were cut using a Biodot Guillotine cutter. Then, 20 ng of UCNP reporters was dried onto the conjugate pad of each of the strips in 50 mM Na-carbonate buffer, pH 9.6, containing 0.5% Tween-20, and 0.5% BSA, 0.06% bovine gamma globulin, 0.05% NaN3 and 5% sucrose. The strips were dried for one hour at +35 °C, protected from humidity. The strips were placed in a plastic housing before assaying the samples.

### 2.4. UCNP-LFA Procedure

First, 10 µL of sample was added into the sample inlet of the plastic cassettes, followed by applying 90 µL of chase buffer (10 mM Tris-HCl, pH 8.5, 135 mM NaCl, 0.5% Tween-20, 1% BSA and 0.06% bovine gamma globulin). After 30 min, the test and control line signals were measured with an Upcon reader device (Labrox Oy, Turku, Finland). The UCNP reporters were excited with a 980-nm infrared laser and the upconversion luminescence emission was detected at a 550-nm wavelength.

### 2.5. Dry Reagent UCNP-LFA Development

Antigen dispensing compatibility on different nitrocellulose membranes with wicking rates of 100–220 s/4 cm was studied. Nitrocellulose membranes used in the study are listed in Table 2. Two different liquid-dispensing instruments (SciSpotter, Scienion AG, and Linomat 5, Camag, Muttenz, Switzerland) were used for line preparation and the test lines dispensed with these instruments were compared in the UCNP-LFA. Dry-conjugate release from the conjugate pad was studied by drying the r-HIV1-env/r-HIV2-env UCNP conjugates in the presence of different additives to the glass fiber conjugate release pad. Additives included 5% sucrose as received or supplied with either 1% Tween-20, 1% Triton-X-100 or 0.9% NaCl. Furthermore, the effect of replacing sucrose with trehalose was studied. For the dry reagent UCNP-LFA, the optimal running buffer pH and composition was studied. For assay development, anti-gp41 rabbit serum (ProSpec-Tany TechnoGene Ltd.) diluted in goat serum was used as a model analyte.

### 2.6. LFA Performance Evaluation

The developed assay was evaluated using the clinical samples and sample panels described above. Total sample numbers used for the assay performance evaluation were 145 anti-HIV-positive and 309 anti-HIV-negative samples.

To compare the performance of the developed UCNP-LFA to a conventional LFA, a set of 100 available anti-HIV-positive and 100 randomly selected anti-HIV-negative samples were tested with the Alere HIV Combo LFA according to the manufacturer’s instructions. The visual results were interpreted by two individuals who were blinded for the reference assay results and the test strips were photographed. The results of only the antibody lines were used to compare with the UCNP-LFA.

The cutoff value for UCNP-LFA was determined based on receiver operating characteristic (ROC) analysis executed using SAS JMP Pro 14 statistics software. To calculate the signal-to-cutoff ratio (S/Co), the photoluminescence signal obtained from each sample with peak detection was divided by the cutoff. Samples with S/Co values ≥ 1 were considered reactive.

## 3. Results and Discussion

**Figure 2:**
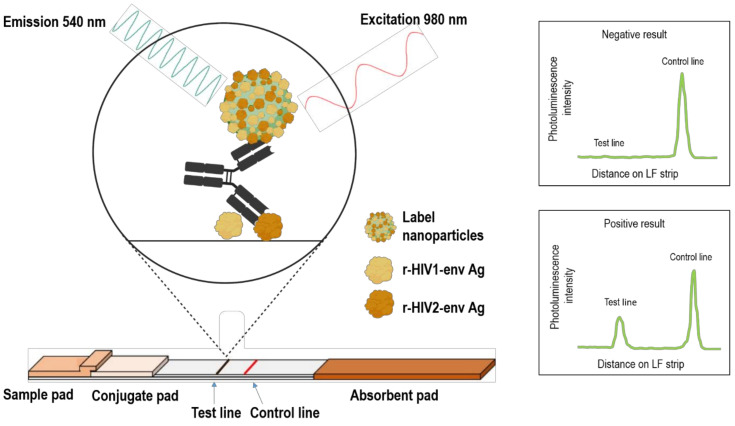
Anti-HIV-1/2 UCNP-LFA strip design and double-antigen bridge assay principle. Anti-HIV antibodies are detected by using recombinant HIV-1 and HIV-2 envelope antigens as captures on the test line as well as coupled to the UCNP surface. UCNPs are excited at 980 nm and the emission peak intensity at 540 nm indicates a negative or positive read-out.

To demonstrate the applicability of UCNP reporter technology for the detection of anti-HIV antibodies, we developed a UCNP-based LFA for the detection of anti-HIV-1/2 antibodies by using the double-antigen bridge assay format, which corresponds to a third-generation anti-HIV assay. The developed assay procedure is simple: addition of 10 µL sample is followed by addition of buffer to the test cartridge including a dry reagent test strip, and the luminescence signals are read 30 min afterwards. The double-antigen bridge format utilizes HIV-1 and -2 envelope glycoproteins described earlier [22,23,24] and the result read-out is based on the test line peak signal intensity originating from the formation of the double-antigen sandwich complex (Figure 2).

### 3.1. Dry Reagent UCNP-LFA Strip Optimization

**Figure 3:**
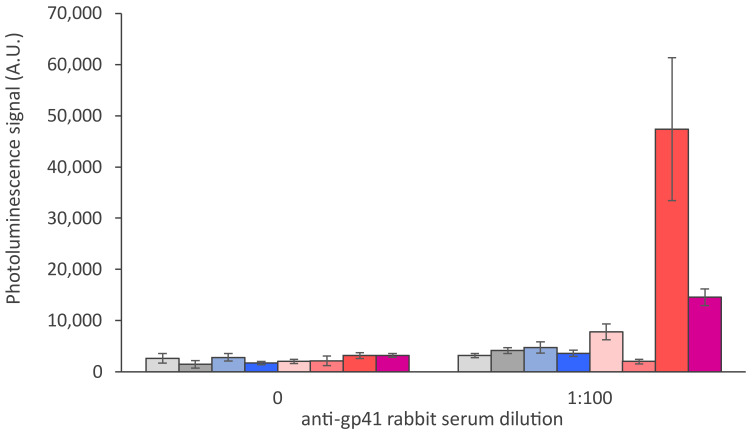
Nitrocellulose membrane comparison in the UCNP-LFA. Eight different nitrocellulose membranes with wicking rates of 100, 120, 125, 140, 150, 180, 200 and 220 s/4 cm (from left to right, respectively; details in Table 2) were compared in the UCNP-LFA with 1:100 dilution of anti-gp41 rabbit serum. The error bars represent the standard deviation among three replicate strips.

**Figure 4:**
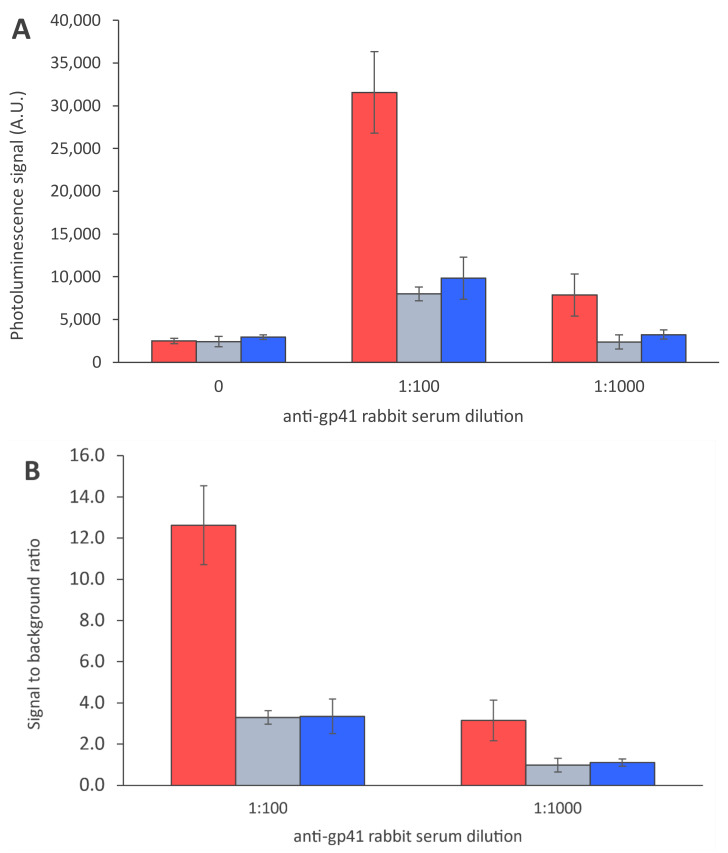
Antigen dispensing on different nitrocellulose membranes. Antigens were dispensed on the following membranes: CNPH-N-SS60 with wicking rate of 200 s/4 cm (red), CNPH-N SS40 with wicking rate of 150 s/4 cm (grey) and CNPF SN12, 5 µm with wicking rate of 220 s/4 cm (blue). The data are shown as test line maximum photoluminescence signals (**A**) and signal-to-background ratios (**B**). The error bars represent the standard deviation among three replicate strips.

The dry reagent assay strip parameters were optimized to enable double-antigen-based anti-HIV antibody detection in the UCNP-LFA format. The capture antigens (r-HIV1-env and r-HIV2-env) were dispensed on different nitrocellulose membranes and the nitrocellulose performance was studied with UCNP-LFA. Membrane performance in the UCNP-LFA is shown in Figure 3. Faster membranes showed reduced detection capability of anti-HIV antibodies. Further comparison of the three best performing membranes is shown in Figure 4. One of the membranes (CNPH-N-SS60) was observed to improve anti-HIV antibody detection in contrast to the two other membranes. Three membranes used in the comparison represented relatively slow wicking rates (150–220 s/4 cm). However, the slow wicking rate was not the only parameter contributing to the membrane performance in the UCNP-LFA, as the slowest membrane did not outweigh the performance of the second slowest membrane CNPH-N-SS60. The membrane selected for an LFA should not only be determined based on the wicking rate but also, e.g., on the protein-binding capacity of the membrane and the membrane surfactant compatibility with the used capture protein.

**Figure 5:**
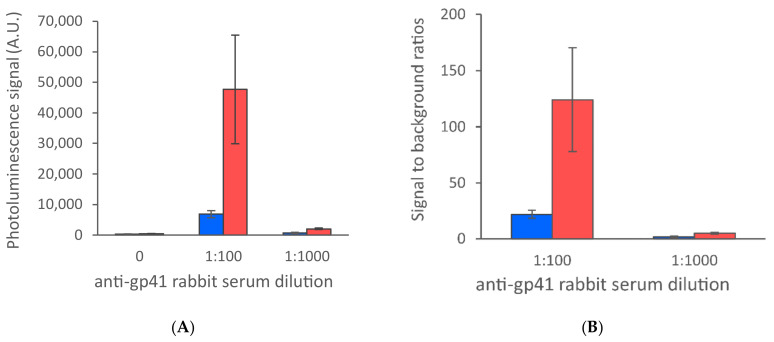
Comparison of liquid-dispensing instruments and line widths. Two different liquid-dispensing instruments (SciSpotter, Scienion AG, and Linomat 5, Camag, Switzerland) were used for line preparation and the test lines dispensed with these instruments were compared in the UCNP-LFA. Thinner lines produced with SciSpotter (red) provided higher signal levels in the presence of anti-HIV antibodies in contrast to the Linomat 5 dispenser (blue). The data are shown as test line maximum photoluminescence signals (**A**) and signal-to-background ratios (**B**). The error bars represent the standard deviation among three replicate strips.

Two different liquid-dispensing instruments were used to apply the test and control lines on the nitrocellulose membranes. In contrast to the Linomat 5 liquid dispenser (Camag), the SciSpotter array dispenser was used to produce thinner lines and study the line width effect on the UCNP-LFA. With the thinner lines produced with the SciSpotter device, higher signals were detected at the test line in the presence of anti-HIV antibodies (Figure 5). Traditionally, an adequate line width is desired with visual LFAs as visual signal development is favored by larger colored areas. However, in case of sensitive detection of UCNP labels, a thinner line may provide an advantage since a higher concentration of the immune complex is achieved on the thinner test line while the measurement instrumentation is still able to precisely detect the UCNP signal from the thin line. With the thinner lines, higher strip-to-strip variation was observed, particularly with higher antibody titers. Higher variation between replicates is associated with high signal levels from individual strips. Furthermore, the variation is relatively smaller with lower antibody concentrations and thus does not result in false negative replicates and does not interfere with the qualitative assay.

**Figure 6:**
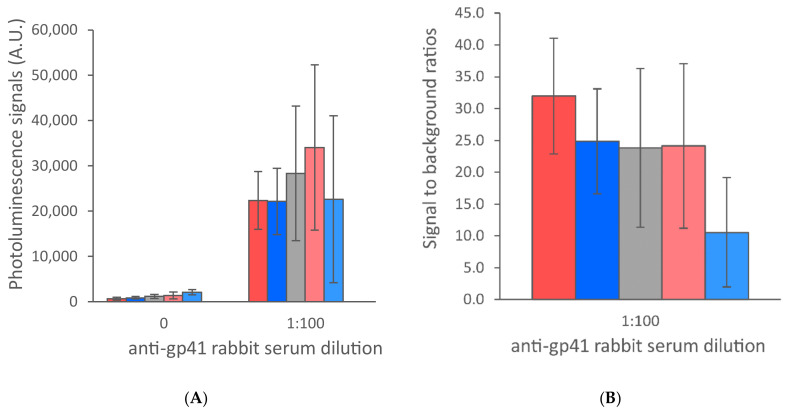
Dry conjugate release from the conjugate pad was studied by drying the r-HIV1-env/r-HIV2-env UCNP conjugates in the presence of different additives to the glass fiber conjugate release pad. Additives included 5% sucrose as received (red) or supplied with either 1% Tween-20 (blue), 1% Triton-X-100 (grey) or 0.9% NaCl (light red) and 5% trehalose as received (light blue). The data are shown as test line maximum photoluminescence signals (**A**) and signal-to-background ratios (**B**). The error bars represent the standard deviation among three replicate strips.

Drying of the r-HIV1-env- and r-HIV2-env-conjugated UCNP reporters on the glass fiber conjugate pad was optimized by using different additives in the reported drying solution. With r-HIV1/2-env antigen-conjugated UCNPs, sucrose over trehalose as a drying additive was observed to provide more consistent results in the LFA in terms of reduced strip-to-strip variation and lower test line background level, providing better capability to distinguish test line signals from the background (Figure 6). Supplementing reporter drying solution with other additives such as Triton-X-100, Tween-20 or NaCl increased the strip-to-strip variation.

**Figure 7:**
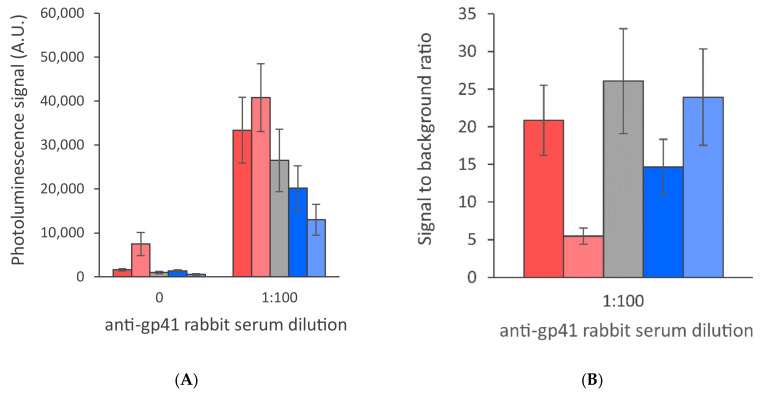
Running buffer comparison. Buffers with pH 7.5 (red), pH 7.5 supplemented with 500 mM NaCl (light red), pH 8.5 (grey), pH 9 (blue) and pH 9.6 (light blue) were compared in the UCNP-LFA. The data are shown as test line maximum photoluminescence signals (**A**) and signal-to-background ratios (**B**). The error bars represent the standard deviation among three replicate strips.

The optimal reaction pH and running buffer composition was determined for the developed UCNP-LFA. Buffers with pH range of 7.5–9.6 were tested in the LFA. In addition, the effect of high salt concentration in the reaction was studied. The results are shown in Figure 7. The increase in buffer pH led to a decrease in the signal levels in the presence of anti-HIV antibodies. High salt running buffer caused an increase in the background signal levels. For the optimal reaction between the r-HIV1/2-env antigens and anti-HIV antibodies, pH < 9 was favorable, whereas the UCNP flow properties on the nitrocellulose membrane were observed to improve with increasing running buffer pH (data not shown).

**Figure 8:**
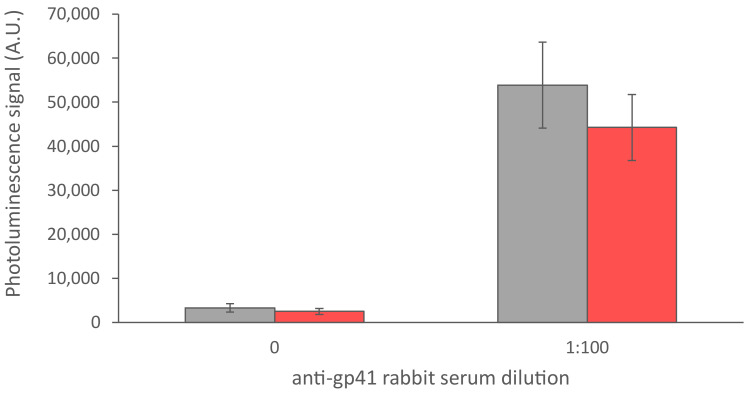
Comparison between anti-gp41 rabbit serum diluted in serum (grey) and in whole blood (red). The error bars represent the standard deviation among three replicate strips.

The UCNP-LFA compatibility with whole blood samples was tested by comparing anti-gp41 rabbit serum spiked serum and whole blood samples (Figure 8). However, the patient samples and sample panels used in this study were only available as plasma and serum. The developed UCNP-LFA should be further evaluated with freshly drawn whole blood samples. The use of finger-prick whole blood in rapid testing of infectious diseases is highly preferred as the sample as such does not require pre-treatment and can be directly applied to the test strip, thus facilitating the testing procedure.

### 3.2. Overall Assay Performance

**Figure 9:**
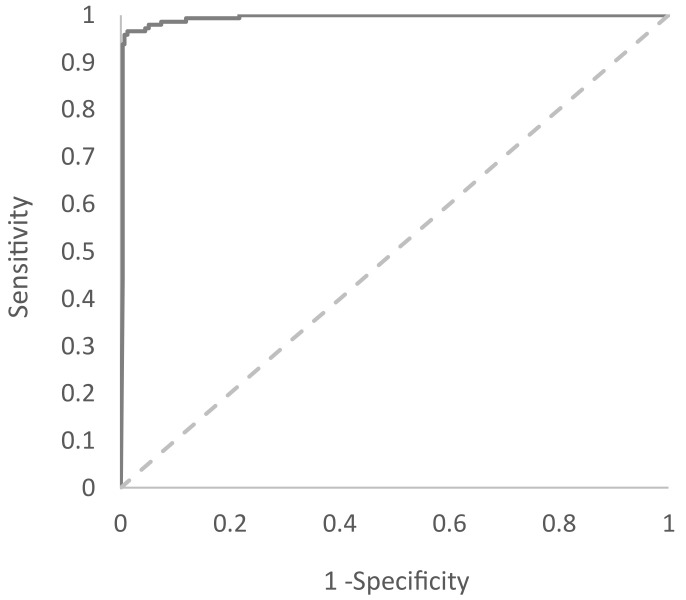
Diagnostic accuracy of Anti-HIV-1/2 UCNP-LFA. Receiver operating characteristic (ROC) of the UCNP-LFA was calculated based on 145 anti-HIV-positive and 309 anti-HIV-negative samples. The area under the curve (AUC) is 0.994.

The developed UCNP-LFA was evaluated with 145 anti-HIV antibody-positive and 309 anti-HIV antibody-negative plasma and serum samples. The UCNP-LFA showed 96.6% (95% CI: 92.1–98.8%) sensitivity and 98.7% (95% CI: 96.7–99.7% specificity (Table 3) based on the ROC analysis (Figure 9). The UCNP-LFA performance was compared to that of the conventional LFA with visual detection by using a limited subset of the samples (*n* = 200). In total, 100 randomly selected anti-HIV-negative samples as well as 100 available anti-HIV-positive samples were run with the conventional test. The sensitivity and specificity of the conventional test were 98.0% (95% CI: 93.0–99.8%) and 100% (95% CI: 96.4–100%), respectively. The same set of samples was used for calculating the sensitivity and specificity of the UCNP-LFA. Within this limited set of samples, those were 95.0% (95% CI: 88.7–98.4%) and 100% (95% CI: 96.4–100%), respectively.

As the data were further analyzed, it was observed that the limited sensitivity of the UCNP-LFA was mainly due to the weaker capability of the assay to detect anti-HIV-2 antibodies. The sensitivities for the detection of HIV-1 (*n* = 83) were 96.4% (95% CI: 89.8–99.3%) for the UCNP-LFA and 97.6% (95% CI: 91.6–99.7%) for the conventional LFA. In contrast, the sensitivities for the detection of HIV-2 (*n* = 17) were 88.2% (95% CI: 63.6–98.5%) and 100% (95% CI: 80.5–100%), respectively (Table 3).

The UCNP-LFA detected 80 out of 83 anti-HIV-1-positive samples whereas the conventional LFA detected 81 samples. With anti-HIV-2-positive samples, UCNP-LFA detected 15 out of 17 samples, when the conventional LFA detected all the samples.

Among the samples tested with both assays, UCNP-LFA and Alere HIV Combo, some of the samples resulted in discrepant results between these two assays. The discrepant results are presented in Table 4. The UCNP-LFA had false negative results somewhat more frequently among these 14 samples. However, the conventional LFA had six equivocal read-outs. Five of the six were still considered true positive when calculating the assay performance. However, the persons interpreting the results were highly trained and the conditions were optimal to read faint lines. For these results, there was a high risk of a false interpretation of the result in case of an untrained interpreter, poor lighting or both.

The performance of the developed UCNP-LFA was very close to that of the conventional LFA, which was expected since antibody detection may be hard to significantly improve due to typically high antibody titers. However, it was observed that the UCNP-LFA had reduced detection capability for anti-HIV-2-type antibodies. This may be due to the weaker binding affinity between r-HIV2-env antigens and the target antibodies in comparison to the binding between r-HIV1-env antigens and anti-HIV-1 antibodies. In the future, the clinical sensitivity of the UCNP-LFA for the detection of HIV-2 antibodies could be improved by either replacing or modifying the r-HIV-2env antigen.

### 3.3. Assay Performance with Commercial Sera Panels

Anti-HIV-1 Mixed Titer Panel (Table 5) performance was very similar between the two LFAs. Anti-HIV1 Low Titer Panel (Table 6) UCNP-LFA detected 2 samples fewer than the conventional LFA. However, one of the samples was equivocal with the conventional LFA. With the HIV-1 Early Infection Performance Panel (Table 7), the LFAs had a similar performance except for panel member 0800-297-16. With the AccuSet HIV-1/2 Performance Panel (Table 8), the LFAs performed equally. With the AccuSet HIV-1 p24 Performance Panel (Table 9), the UCNP-LFA was able to detect two extremely low positive samples which remained undetected by the conventional LFA.

### 3.4. UCNP-LFA Feasibility for Infectious Disease Testing

In this study, we used UCNPs as reporters in the LFA. The UCNP signal can be read with a portable reader device. This provides unambiguous results, and therefore, the results’ interpretation is not dependent on the training level of the interpreter or the circumstances. In addition, data archiving and transfer are also simpler with this approach. Approaches to detect conventional visual labels quantitatively have also been developed due to various application requirements [25].

Visual read-out is popular in LFAs since it is user-friendly and does not necessarily require additional instrumentation. Visual labels used in LFAs are typically colloidal cold nanoparticles, though other types of colored labels such as carbon nanoparticles can be used. Advantages of colloidal cold particles are easy synthesis, simple bioconjugation procedure, intense color facilitating visual read-out without color development process and the stability of the label in liquid and dried forms [26,27]. When considering using luminescent labels such as UCNPs in LFAs instead of visual labels, similar advantages can be seen. The UCNPs are stable in liquid and dry forms. Biomolecules are covalently coupled to the UCNP surface. UCNPs do not photo-bleach and can be excited several times [28]. This also enables storage and transport of assayed LFA strips, and the strips can be measured later, which can give an advantage in remote locations. Visual labels typically have a time frame for the read-out. Requirement for the reader instrument adds absolute cost for the use of UCNP reporters; however, the optics required for upconversion luminescence detection can be provided in a miniaturized and cost-effective format, e.g., as described in the literature [16,17]. Furthermore, the possible reader device connection to electronic healthcare services may provide relative cost savings for the healthcare system and improve access to better healthcare by enabling better patient management regardless of geographic and socioeconomic limitations [29]. In addition, the same reader platform could be used for reading other UCNP-based tests to expand the testing capacity at the remote health care site.

Typically, rapid tests targeted for use in LMICs are recommended to follow the ASSURED criteria established by the WHO [30]. The acronym ASSURED stands for affordable, sensitive, specific, user-friendly, rapid and robust, equipment-free and deliverable. The developed UCNP-LFA can use low-cost portable battery-operated reader devices for the read-out. This kind of reader technology can be implemented in remote locations in LMICs. A reader provides opportunities for on-site result analysis or remote data transfer to a centralized facility for more detailed analysis in resource-limited settings [29]. Furthermore, the strips can be run and transported for reading in case the reader cannot be taken to the location due to the above-described properties of UCNPs. The assay procedure is simple and user-friendly, only requiring addition of a sample, addition of a buffer and reading with the instrument. The turn-around time (TAT) of the developed test was 30 min, thus being within the WHO recommendation of 30 min or less [19]. The TATs of the LFA-based RDTs for HIV detection vary from 15 to 25 min [31]. The time frame for the read-out of the Alere HIV Combo rapid LFA test is from 20 to 40 min.

The most significant advantage of using UCNP reporters in LFA is the improvement in analytical sensitivity due to the unique photoluminescence properties of the UCNPs. UCNPs upconvert long-wavelength infrared excitation light into shorter-wavelength higher-energy visible light through multiphoton excitation and long lifetime phosphorescent emission. Intense infrared excitation can be provided with a small inexpensive infrared laser and the resulting luminescence with a wide anti-Stokes shift can be easily separated from the autofluorescence caused by the test or sample materials [28]. These properties can allow the development of highly sensitive UCNP-LFA tests for antigen markers. However, the direct advantage of improved analytical sensitivity is more limited when testing for an antibody response. Previously, we have described the development of sensitive rapid assays for the detection of *Plasmodium falciparum* malaria [32] and hepatitis B virus surface antigen [33] by using the UCNP-LFA platform, resulting in a 15- to 250-fold improvement in analytical detection sensitivity in contrast to the conventional rapid tests. Other research groups have reported sensitivity improvement achieved by using UCNP detection technology in testing of, e.g., antibiotic compounds [34] and myoglobin [35].

## 4. Conclusions

The developed UCNP-LFA for anti-HIV antibodies allows the detection of HIV infection with the UCNP reader system, and the detection capacity of the measurement system can be further expanded to detect other infections prevalent in LMICs such as malaria and hepatitis B [32,33]. Use of the same detection technology in a variety of tests, e.g., a test panel for infectious diseases including antigen and antibody markers, allows the use of a single measurement instrument with automated result interpretation, archiving and transfer of results, which can provide cost savings.

## Figures and Tables

**Table 1:** Clinical samples used in the evaluation of UCNP-LFA.

**Samples/Sample Panel**	**Purchased from**	**Sample Matrix**	**Anti-HIV-1+**	**Anti-HIV-2+**	**NEG**
HIV disease state samples	Labquality Oy, Finland	Plasma	25	6	22
Anti-HIV-1 Mixed Titer (0800-0303) (PRB205)	SeraCare Life Sciences Inc., USA	Plasma	16	0	1
Anti-HIV-1 Low Titer (0800-0301)		Plasma	13	0	2
HIV-1 Early Infection Performance (0800-0297)		Plasma	11	0	12
AccuSet HIV-1 p24 perf. Panel (0800-0362)		Plasma	8	0	5
Accuset HIV-1/2 perf. Panel (0800-0380)		Plasma	6	6	1
HIV-1/2 WW perf. Panel (PRZ206)		Plasma	6	6	1
Viral Co-infection Panel (PCA201)		Plasma	16	0	7
Anti-HIV positive disease state samples	Biomex GmbH, Germany	Serum	13	5	0
Presumed healthy samples	Turku University of Applied Sciences, Finland	Serum	0	0	100
**Samples/Sample Panel**	**Obtained from**	**Sample Matrix**	**Anti-HIV-1+**	**Anti-HIV-2+**	**NEG**
Routine-tested clinical samples negative for anti-HIV	Department of Virology, University of Turku, Finland	Serum/plasma	0	0	216
Routine-tested clinical samples positive for anti-HIV		Serum	15	0	1

**Table 2:** Nitrocellulose membranes studied in the UCNP-LFA.

Nitrocellulose Membrane	Supplier	Wicking Rate (s/4 cm)
CNPC SS12, 15 µm	Advanced Microdevices (India)	100
CNPC SS12, 12 µm	Advanced Microdevices (India)	120
CNPF SN12, 10 µm	Advanced Microdevices (India)	125
CNPC SS12, 10 µm	Advanced Microdevices (India)	140
CNPH-N SS40	Advanced Microdevices (India)	150
HF180	Millipore Corporation (USA)	180
CNPH-N SS60	Advanced Microdevices (India)	200
CNPF SN12, 5 µm	Advanced Microdevices (India)	220

**Table 3:** Assay performances with clinical patient samples compared between the developed Anti-HIV-1/2 UCNP-LFA and Alere HIV Combo.

	Anti-HIV UCNP-LFA	Alere HIV Combo (*n* = 200)	Anti-HIV UCNP-LFA (*n* = 200)
Number of tested positive samples	145	100	100
Anti-HIV-1	123	83	83
Anti-HIV-2	22	17	17
Number of tested negative samples	309	100	100
Number of samples with agreeing results			
True positive	140	98	95
Anti-HIV-1		81	80
Anti-HIV-2		17	15
True negative	305	100	100
Number of samples with disagreeing result			
False positive	4	0	0
False negative	5	2	5
Anti-HIV-1		2	3
Anti-HIV-2		0	2
Total number of samples	449	10 0	100
Sensitivity	96.6% (95% CI: 92.1–98.8%)	98.0% (95% CI: 93.0–99.8%)	95.0% (95% CI: 88.7–98.4%)
Anti-HIV-1 detection	-	97.6% (95% CI: 91.6–99.7%)	96.4% (95% CI: 89.8–99.3%)
Anti-HIV-2 detection	-	100% (95% CI: 80.5–100%)	88.2% (95% CI: 63.6–98.5%)
Specificity	98.7% (95% CI: 96.7–99.7%)	100% (95% CI: 96.4–100%)	100% (95% CI: 96.4–100%)

**Table 4:** Discrepant results between the Alere HIV Combo test and the developed Anti-HIV-1/2 UCNP-LFA.

Sample ID	Panel	Status ^1^	UCNP-LFA (S/Co) ^2^	Alere HIV Combo Result ^3^
#1	Disease state sample	NEG	0.2	+/-
#2	Disease state sample	anti-HIV-1+	0.9	+
#3	Disease state sample	anti-HIV-1+	3.2	+/-
#4	Disease state sample	anti-HIV-1+	1.3	+/-
#5	Disease state sample	anti-HIV-1+	0.7	+/-
#6	Disease state sample	anti-HIV-1+	0.6	+
#7	Disease state sample	anti-HIV-2+	0.5	+
#8	Disease state sample	anti-HIV-2+	0.7	+
0800-0301-03	Anti-HIV-1 low titer panel	NEG	0.9	+/-
0800-0301-04	Anti-HIV-1 low titer panel	anti-HIV-1+	0.8	+
0800-0297-16	HIV-1 early infection performance panel	anti-HIV-1+	0.8	+
0800-0362-02	Accuset HIV-1 p24 performance panel	anti-HIV-1+	1.2	-
0800-0362-05	Accuset HIV-1 p24 performance panel	anti-HIV-1+	1.1	-
0800-0362-11	Accuset HIV-1 p24 performance panel	anti-HIV-1+	0.6	+/-

^1^ Sample status information for the disease state samples was obtained from the supplier. Reference data for panel samples are provided in Tables 1, 6, 7 and 9. ^2^ For UCNP-LFA, signal-to-cutoff (S/Co) ratios ≥ 1.0 are considered reactive. ^3^ Tests were run in our laboratory. Interpretation: +++ strong visible test line; ++ visible test line; + faint visible test line; +/- equivocal; - no visible test line.

**Table 5:** Anti-HIV-1 Mixed Titer Panel (0800-0303) (PRB205).

Member	Abbott HIV 1/2 rDNA EIA ^1^	Bio-Rad HIV 1/2 Plus O ^1^	OraQuick ADVANCE ^1^	UCNP-LFA ^2^	Alere HIV Combo ^3^
0800-303-1	7.2	>10.1	Neg	1.5	+
0800-303-2	18.4	>10.1	Pos	12.4	++
0800-303-4	>18.5	>10.1	Pos	12.9	+++
0800-303-5	>18.5	>10.1	Pos	8.4	++
0800-303-7	6.5	>10.1	Neg	3.1	+
0800-303-9	>18.5	>10.1	Pos	16.3	+++
0800-303-10	>18.5	>10.1	Pos	8.5	+
0800-303-11	>18.5	>10.1	Pos	8.7	+++
0800-303-12	6.9	2.5	Neg	2.6	+
0800-303-13	>18.5	>10.1	Pos	6.8	++
0800-303-14	0.2	0.4	Neg	0.3	-
0800-303-15	10.8	>10.1	Pos	3.5	++
0800-303-16	>18.5	>10.1	Pos	8.0	++
0800-303-17	7.2	>10.1	Pos	4.5	++
0800-303-18	10.9	>10.1	Neg	1.2	++
0800-303-19	13.1	>10.1	Pos	2.8	++
0800-303-20	>18.5	>10.1	Pos	11.0	+++

Numerical results are the S/Co ratios. ^1^ Results from the panel data sheet. S/Co ratios ≥ 1.0 are reactive. ^2^ For UCNP-LFA, S/Co ratios ≥ 1.0 are considered reactive. ^3^ Tests were run in our laboratory. Interpretation: +++ strong visible test line; ++ visible test line; + faint visible test line; +/- equivocal; - no visible test line.

**Table 6:** Anti-HIV-1 Low Titer Panel (0800-0301).

Member	Abbott HIV 1/2 rDNA EIA ^1^	Bio-Rad HIV 1/2 Plus O ^1^	OraQuick ADVANCE ^1^	Inverness Determine^®^ HIV-1/2 Ag/Ab Combo ^1^		UCNP-LFA ^2^	Alere HIV Combo ^3^
0800-301-01	4.1	>10.9	Neg	Pos	Neg	2.1	+
0800-301-02	0.7	>10.9	Neg	Pos	Pos	0.8	-
0800-301-03	0.6	8.5	Neg	Neg	Neg	0.9	+/-
0800-301-04	2.1	>10.9	Neg	Neg	Pos	0.8	+
0800-301-07	0.7	1.3	Neg	Neg	Neg	1.0	+
0800-301-08	0.1	0.1	Neg	Neg	Neg	0.8	-
0800-301-10	10.3	>10.9	Neg	Neg	Pos	3.6	++
0800-301-11	7.4	>10.9	Pos	Neg	Pos	3.4	++
0800-301-12	11.8	>10.9	Pos	Neg	Pos	9.6	++
0800-301-13	0.5	0.3	Neg	Neg	Neg	0.4	-
0800-301-14	10.1	>10.9	Neg	Pos	Pos	4.1	++
0800-301-15	7.2	>10.9	Pos	Neg	Pos	8.0	++
0800-301-16	6.1	>10.9	Neg	Pos	Pos	3.2	++
0800-301-17	2.2	>10.9	Neg	Pos	Pos	3.9	+
0800-301-19	16.3	>10.9	Pos	Neg	Pos	12.2	+++

Numerical results are the S/Co ratios. ^1^ Results from the panel data sheet. S/Co ratios ≥ 1.0 are reactive. ^2^ For UCNP-LFA, S/Co ratios ≥ 1.0 are considered reactive. ^3^ Tests were run in our laboratory. Interpretation: +++ strong visible test line; ++ visible test line; + faint visible test line; +/- equivocal; - no visible test line.

**Table 7:** HIV-1 Early Infection Performance Panel (0800-0297).

Member	Bio-Rad GenSys HIV 1/2 Plus O EIA ^1^	OraQuick ADVANCE^® 1^	Bio-Rad Multispot HIV-1/2 Rapid Test ^1^		UCNP-LFA ^2^	Alere HIV Combo ^3^
0800-297-1	0.05	Neg	Neg	Neg	0.8	-
0800-297-2	0.03	Neg	Neg	Neg	0.6	-
0800-297-3	0.04	Neg	Neg	Neg	0.5	-
0800-297-4	0.03	Neg	Neg	Neg	0.8	-
0800-297-5	0.05	Neg	Neg	Neg	0.8	-
0800-297-6	0.09	Neg	Neg	Neg	0.5	-
0800-297-7	0.05	Neg	Neg	Neg	0.5	-
0800-297-8	0.03	Neg	Neg	Neg	0.6	-
0800-297-9	0.04	Neg	Neg	Neg	0.9	-
0800-297-10	0.04	Neg	Neg	Neg	0.9	-
0800-297-11	0.03	Neg	Neg	Neg	0.4	-
0800-297-12	0.04	Neg	Neg	Neg	0.8	-
0800-297-13	3.4	Neg	Pos	Neg	2.9	++
0800-297-14	1.8	Neg	Neg	Neg	3.6	++
0800-297-15	1.2	Neg	Neg	Neg	0.6	-
0800-297-16	2.0	Neg	Neg	Neg	0.8	+
0800-297-17	3.5	Weak Pos	Pos	Neg	2.8	++
0800-297-18	3.6	Weak Pos	Pos	Neg	4.5	+
0800-297-19	3.6	Neg	Neg	Neg	1.4	++
0800-297-20	3.6	Neg	Pos	Neg	1.8	+
0800-297-21	3.6	Pos	Pos	Neg	8.2	++
0800-297-22	3.7	Pos	Pos	Neg	6.0	+
0800-297-23	3.7	Strong Pos	Pos	Neg	3.7	++

Numerical results are the S/Co ratios. ^1^ Results from the panel data sheet. S/Co ratios ≥ 1.0 are reactive. ^2^ For UCNP-LFA, S/Co ratios ≥ 1.0 are considered reactive. ^3^ Tests were run in our laboratory. Interpretation: +++ strong visible test line; ++ visible test line; + faint visible test line; +/- equivocal; - no visible test line.

**Table 8:** AccuSet HIV-1/2 Performance Panel (0800-0380).

Member	HIV Type	Avioq HIV-1 Microelisa ^1^	Bio-Rad GenSys HIV-2 EIA ^1^	OraQuick ADVANCE ^1^	UCNP-LFA ^2^	Alere HIV Combo ^3^
0800-380-01	1	8.8	0.9	Pos	15.9	+++
0800-380-02	1	8.7	1.0	Pos	27.1	+++
0800-380-03	1	8.2	0.2	Pos	12.0	+++
0800-380-04	1	8.5	0.7	Pos	14.9	+++
0800-380-05	1	6.7	0.6	Pos	11.4	+++
0800-380-06	1	9.1	1.3	Pos	19.1	++
0800-380-07	Neg	0.2	0.2	Neg	0.8	-
0800-380-08	2	2.2	16.3	Pos	2.8	+++
0800-380-09	2	1.7	19.2	Pos	2.8	+++
0800-380-10	2	2.9	15.7	Pos	4.0	++
0800-380-11	2	1.1	14.1	Pos	2.9	++
0800-380-12	2	0.9	14.2	Pos	3.0	++
0800-380-13	2	0.6	11.2	Pos	5.6	+++

Numerical results are the S/Co ratios. ^1^ Results from the panel data sheet. S/Co ratios ≥ 1.0 are reactive. ^2^ For UCNP-LFA, S/Co ratios ≥ 1.0 are considered reactive. ^3^ Tests were run in our laboratory. Interpretation: +++ strong visible test line; ++ visible test line; + faint visible test line; +/- equivocal; - no visible test line.

**Table 9:** AccuSet HIV-1 p24 Performance Panel (0800-0362).

Member	DiaSorin LIAISON XL ^1^	Bio-Rad GenSys HIV1/2 PLUS O EIA ^1^	Avioq HIV-1 Microelisa ^1^	OraQuick ADVANCE ^1^	UCNP-LFA ^2^	Alere HIV Combo ^3^
0800-362-1	25.7	13.3	3.2	Pos	5.9	++
0800-362-2	1.4	2.6	0.3	Neg	1.2	-
0800-362-3	0.2	0.8	0.2	Neg	0.8	-
0800-362-4	39.4	13.3	1.6	Neg	14.3	++
0800-362-5	1.1	0.2	0.3	Neg	1.0	-
0800-362-6	0.3	0.8	0.3	Neg	0.4	-
0800-362-7	66.8	13.3	4.7	Pos	9.9	+++
0800-362-8	11.8	13.3	1.2	Neg	5.0	+
0800-362-9	0.2	0.2	0.3	Neg	0.5	-
0800-362-10	0.3	0.1	0.3	Neg	0.4	-
0800-362-11	5.0	5.3	0.3	Neg	0.6	-
0800-362-12	59.8	13.3	5.8	Pos	10.0	+++
0800-362-13	0.3	0.1	0.3	Neg	0.8	-

Numerical results are the S/Co ratios. ^1^ Results from the panel data sheet. S/Co ratios ≥ 1.0 are reactive. ^2^ For UCNP-LFA, S/Co ratios ≥ 1.0 are considered reactive. ^3^ Tests were run in our laboratory. Interpretation: +++ strong visible test line; ++ visible test line; + faint visible test line; +/- equivocal; - no visible test line.

## Data Availability

Data is contained within the article.
